# Exceptions to the rule of informed consent for research with an intervention

**DOI:** 10.1186/s12910-016-0092-6

**Published:** 2016-02-06

**Authors:** Susanne Rebers, Neil K. Aaronson, Flora E. van Leeuwen, Marjanka K. Schmidt

**Affiliations:** Division of Psychosocial Research and Epidemiology, The Netherlands Cancer Institute, Amsterdam, The Netherlands; Division of Molecular Pathology, The Netherlands Cancer Institute, Postbus 90203, 1006 BE Amsterdam, The Netherlands

**Keywords:** Informed consent, Consent waiver, Research ethics

## Abstract

**Background:**

In specific situations it may be necessary to make an exception to the general rule of informed consent for scientific research with an intervention. Earlier reviews only described subsets of arguments for exceptions to waive consent.

**Methods:**

Here, we provide a more extensive literature review of possible exceptions to the rule of informed consent and the accompanying arguments based on literature from 1997 onwards, using both *Pubmed* and *PsycINFO* in our search strategy.

**Results:**

We identified three main categories of arguments for the acceptability of a consent waiver: data validity and quality, major practical problems, and distress or confusion of participants. Approval by a medical ethical review board always needs to be obtained. Further, we provide examples of specific conditions under which consent waiving might be allowed, such as additional privacy protection measures.

**Conclusions:**

The reasons legitimized by the authors of the papers in this overview can be used by researchers to form their own opinion about requesting an exception to the rule of informed consent for their own study. Importantly, rules and guidelines applicable in their country, institute and research field should be followed. Moreover, researchers should also take the conditions under which they feel an exception is legitimized under consideration. After discussions with relevant stakeholders, a formal request should be sent to an IRB.

## Background

Despite the widespread agreement on the legal and ethical appropriateness of the general rule of informed consent for human research with an intervention, there are examples of very specific situations in which there are reasons to make an exception. An example is health research that can only be conducted on incapacitated patients, such as resuscitation research [1].

Over the years, informed consent requirements have undergone some notable changes. The Nuremberg Code was written in response to Nazi war crimes, and stated that informed consent for research is ‘absolutely essential’. An important change in this statement was introduced with the Declaration of Helsinki in 1964, which also allowed proxy consent from a relative in case the subject was unable to make this decision. Policy surrounding waiving consent remains a topic of debate within the scientific literature. Following publication in 1997 of two papers were published, describing studies in which no informed consent was asked [2, 3], the Editor of BMJ invited readers to share their viewpoint on whether publishing these articles was the right decision [4]. This invitation led to the largest volume of correspondence on any specific topic in the history of *BMJ*. The many recent papers on this topic (e.g. [5–7]) in various scientific journals, indicate that this debate is still ongoing.

In the context of an earlier intervention study that we conducted comparing three different consent procedures for the use of residual tissue for scientific research [8], we perused the literature for examples of other studies in which consent was waived, and the conditions under which such a waiver was applied. Most of the literature that we found was focused on only one subset of reasons, or reasons given for only one specific type of research in which the informed consent requirement can be waived. For example, Biros et al [5] provided an overview of conditions under which the American Food and Drug Administration (FDA) permits research in medical emergency circumstances without consent. Giraudeau et al [9], in a review of cluster randomized trials, found that less than 5 % of such trials explicitly stated that no individual level informed consent was required. Other papers have focused on reasons not to ask informed consent in the control group of a randomized controlled trial, commonly known as Zelen’s design or a prerandomization design [10].

To the best of our knowledge, no paper has yet provided a more comprehensive review of the range of arguments and circumstances under which the informed consent requirement in intervention research might be waived. We believe that such a review can serve as an important source document for researchers, institutional review boards, and policy makers involved in establishing the legal and ethical standards of research with human subjects. The discussion about when informed consent is necessary is not recent (e.g. [11]). However, societal developments lead to new insights, and changes in research interests lead to new discussions. Therefore, our aim is to provide a review of contemporary reasons to waive informed consent. For that reason, we reviewed the literature starting from the discussion in *BMJ*. In the discussion, we also reflect on the arguments brought forward in the reviewed papers.

## Methods

We reviewed the literature cited in *Pubmed* and *PsycINFO*, from April 1997, the year in which the discussion of exceptions to informed consent was started in *BMJ,* to September 2013. We searched *Pubmed.gov* using the following search terms: ((("informed consent"[MeSH Terms] AND "epidemiologic studies"[MeSH Terms] AND "data collection"[MeSH Terms]) AND (Review[ptyp] OR Randomized Controlled Trial[ptyp] OR Letter[ptyp] OR Clinical Trial[ptyp] OR Journal Article[ptyp])) OR (("clinical trials as topic"[MeSH Terms] AND "informed consent"[MeSH Terms] AND "ethics, research"[MeSH Terms]) AND (Review[ptyp] OR Randomized Controlled Trial[ptyp] OR Clinical Trial[ptyp] OR Journal Article[ptyp])) OR ("informed consent"[MeSH Terms] AND "intervention studies"[MeSH Terms]) OR ("informed consent"[MeSH Terms] AND "epidemiology"[MeSH Terms]) OR (Consent[All Fields] AND waiver[All Fields])) AND (("1997/04/12"[PDAT] : "2013/08/31"[PDAT]) AND English[Language]).

We searched the *PsycINFO* database using the following search terms: ((DE "Informed Consent" AND DE "Data Collection" AND DE "Epidemiology") OR (DE "Informed Consent" AND DE "Clinical Trials" AND DE "Ethics") OR (DE "Informed Consent" AND DE "Intervention") OR (DE "Informed Consent" AND DE "epidemiology") OR (Consent waiver)) AND LA English AND ED 19970412-20130831 AND PT Peer Reviewed Journal.

Doyal and Tobias collected and published in book form all correspondence in *BMJ* regarding the two papers where a waiver of informed consent [12]. We included all letters in this book in this review. When reasons in these letters were cited by other discussants, we only cited the original author.

Eligible articles discussed reasons not to ask informed consent and/or conditions under which an exception to the rule of informed consent was deemed acceptable. We only included reasons and conditions if they applied to informed consent for scientific research with an intervention, including research with a Zelen (prerandomization) design and deferred consent. We excluded research on children and articles addressing reasons to use proxy consent (surrogate consent) from family members or partners instead of patients themselves (e.g. [13]). Importantly, our search strategy was not aimed at identifying papers about the content of the informed consent procedure, such as the completeness or accuracy of information given to participants before the start of an intervention study. For example, there has been much discussion about the use of deception in informed consent about the research goal of a study in psychology (e.g. [14]). Although the content of informed consent forms is important and related to the topic of this review, it is beyond the scope of this paper.

**Fig. 1:**
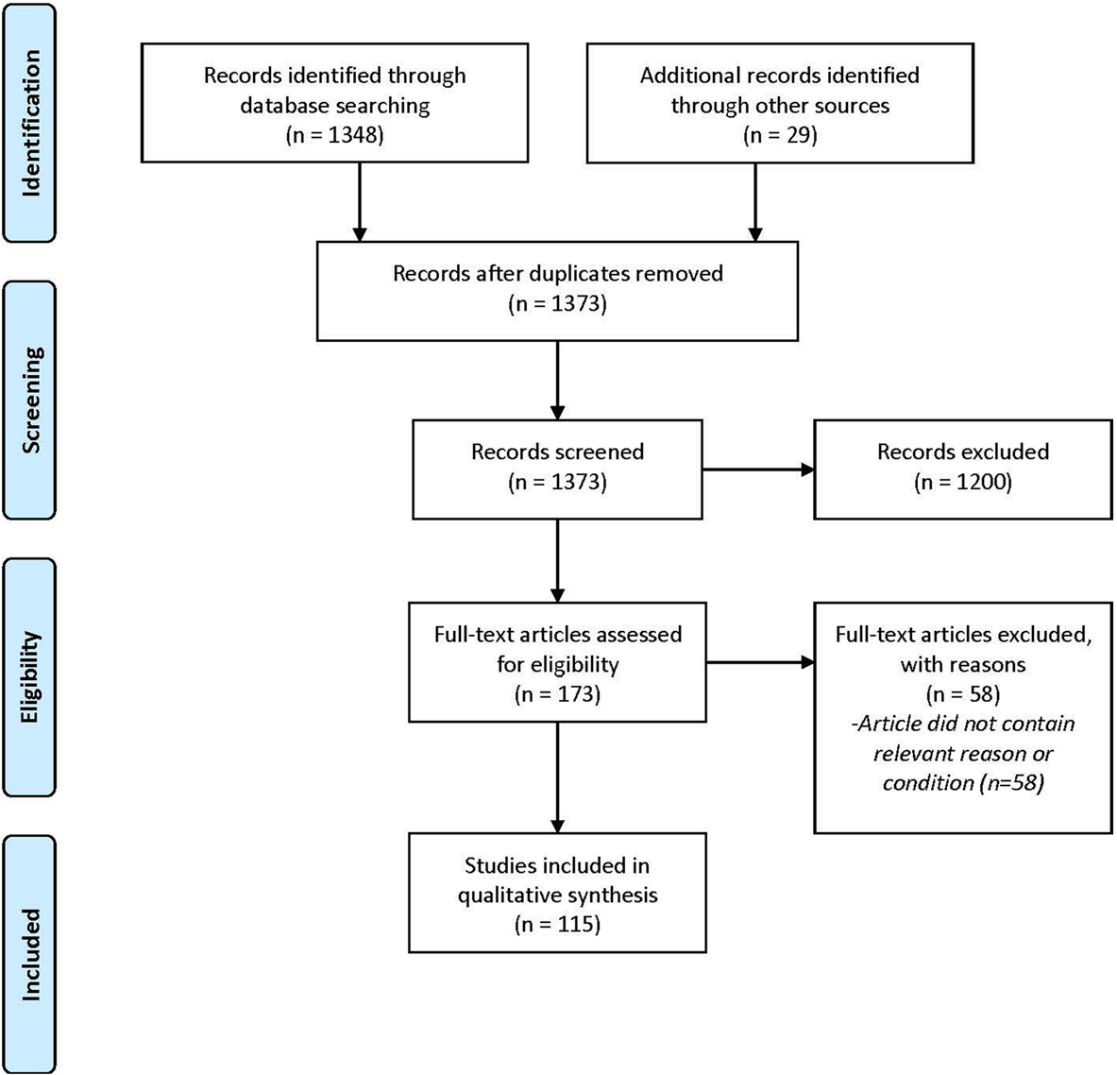
Selection process of eligible studies on reasons to waive informed consent. Search terms are described in the methods section of this paper

The process and decisions on the inclusion and exclusion criteria were discussed with all authors. Then, one of the authors (SR) made a selection of articles based on the title and abstract, and evaluated the selected articles for the inclusion and exclusion criteria (Fig. 1).

## Results

In total 1348 records were identified (Fig. 1). After detailed assessment of eligibility, 115 papers were included in the review. We would note that all arguments given, and the judgements given about these arguments, in the results section do not necessarily reflect the opinions of the authors of this paper, but refer to the opinions and judgements of the authors of the 115 papers included in this review.

Major reasons for exclusion were papers describing research without an intervention (e.g., observational studies), pediatric intervention studies, or papers focusing on reasons to obtain proxy consent. Papers identified through pubmed.gov were not taken into account in the PsycINFO search. From the 115 papers included, we identified three main categories of reasons for waiving the informed consent requirement: (1) decrease of data validity and quality; (2) distress or confusion of participants; and (3) practical problems. Ethical issues play a role in each of the three categories, as an overarching theme. Therefore, we also included a fourth category focusing on ethical reasons or objections to waiving informed consent. We present here all arguments and conditions mentioned in these four categories (the order of the categories and arguments were randomly chosen). For any given argument, all papers mentioning this argument are cited.

### Decrease of data validity and quality

Concerns about the effect of asking informed consent on the quality or validity of outcome data are prevalent in the literature. These concerns are specifically relevant when study outcomes are self-reported, when bias is likely to occur, or when the inclusion rate is highly compromised because of asking informed consent. Many of the examples in this section come from proponents of the Zelen design, in which no consent is asked for randomization [15]. After randomization, researchers ask subjects allocated to the intervention group for informed consent, while subjects in the control group remain in the study without being informed of the randomization procedure.

It has been argued that asking informed consent in studies in which outcomes are self-reported may result in poor data quality, most notably through the ‘Hawthorne effect’ or through ‘resentful demoralization’. The “Hawthorne effect” argument has most typically been raised by investigators working in implementation science where subjects’ behavior and thus the outcome of a study is likely to be influenced significantly by the subjects’ awareness of being part of that study [16–19]. Examples include studies of patient compliance [20], cluster randomized trials in which the effect of offering physicians an intervention to improve uptake of clinical guidelines is studied [21], and studies on the effect of offering a screening tool for the detection of violence at home [22]. In some cases, the consent process is also thought to bias the study outcome [23], for instance when the study is aimed at comparing patients who received information on a topic with patients who were not informed. An informed consent procedure gives patients in the control group information about the topic, which makes them more similar to patients in the intervention group than desirable.” Resentful demoralization” is a term used to describe the impact of the disappointment of being randomized to the control group on self-reported outcomes or outcome behavior [24–27]. Some authors also expect a bias if a preference for the intervention arm influences the validity of a study due to differential drop-out [28]. An example is a study in which researchers provided the drug heroin to addicted persons; many participants withdrew from the study after being randomized to the control group [24, 29]. Institutional Review Board (IRB) approval is often mentioned as a necessity when waiving consent in these cases [16, 18].

Some authors argue that selection bias or participation bias may also decrease the validity of study results [10, 30]. Physicians might, for example, be less likely to ask certain patient groups (e.g., those with a perceived lower intelligence or with social or emotional problems) to consent for research [24]. They further argue, that some patient groups might be less likely to give consent than others [3, 31], such as minority group members [32, 33] or unskilled workers, while other patient groups, especially in emergency research, may not be able to consent at all [34]. In such situations, the outcome of such a study may address the efficacy of treatment among those who consent, while the investigators may be more interested in evaluating the effectiveness of the intervention in a real world setting (i.e., implementation research) [10, 33]. It has been suggested that for a waiver to be acceptable in these cases, all identifiable data should be discarded at the end of the study [35].

Some also expect bias might be introduced when informed consent is asked from all health care workers in a cluster randomized trial [7]. In this case, it has been suggested that an IRB may grant a waiver, provided the researchers provide compelling evidence that the results would be non-interpretable without the waiver, and that a different study design could not overcome this problem. Further, a cluster representative, i.e., a representative of the patient group concerned, should agree to the enrollment of the cluster in the study [21].

A higher inclusion rate has also been mentioned as a reason not to ask for informed consent [10, 24, 36, 37], especially in intensive care trials [37], where inclusion of a relatively small number of patients might otherwise take years. A low inclusion rate could further lead to poor data validity and quality, because it might result in physicians being less accurate in following the study protocol [38]. Moreover, especially in emergency research, where it may be unacceptable to delay start of treatment due to consent procedures, such a delay may also lead to an underestimation of the treatment effect [39, 40].

### Distress or confusion of participants

The second major argument often mentioned in the literature for making an exception to the rule of informed consent in specific situations is that a consent procedure might lead to unnecessary distress or confusion on the participants’ part [16]. The informed consent procedure itself may generate such distress or confusion. For example, severely ill patients who are focused on their treatment and recovery [41], and who may already be quite anxious [42], may find it disturbing to be informed about a study. Similarly, the stressfulness of the medical situation is thought to inhibit potential study participants from grasping the rationale of the study, especially in emergency research [36, 37, 43], for example when patients have suffered an acute myocardial infarction [36]. Others have argued that patients might also be negatively affected by the knowledge that their physician does not know which treatment is best [44, 45].

Being randomized to the control group after being given information about the intervention arm of a study might be especially confusing or disappointing for patients [24, 36, 46]. Some argue that the Zelen design is a more humane way to deal with patients’ expectations of receiving a novel treatment [10], and it may reduce distress associated with knowing that the physician does not always know which treatment is best [45]. Moreover, Homer [24] has argued that use of the Zelen design may also avoid damaging the doctor-patient relationship.

### Practical problems

#### The impossibility of informed consent in emergency research

Emergency research is the most often mentioned example in all contexts described, and it is the most notable research discipline in which practical problems are forwarded as the primary reason why the informed consent requirement should be waived. This is because it is simply impossible to ask (temporarily) incapacitated patients for informed consent [1, 5, 23, 33, 34, 36, 38, 40, 43, 45, 47–95]. Patient populations often mentioned in this context are those with acute head or brain injuries [5, 25, 34, 38, 39, 74, 96], and comatose, unconscious or sedated patients [49, 92], although a much wider range of relevant patient populations has also been named [40, 58, 60, 63, 65, 66, 97–105].

Specifically in the context of emergency research, an often-mentioned reason to waive informed consent is that the main alternative, proxy consent, is often not possible either [87, 99]. This is especially the case when the treatment window is short [1, 5, 23, 25, 32, 34, 36, 37, 43, 56, 57, 61, 63, 65, 68, 69, 77, 80, 96, 98–100, 103, 104, 106]. Others have mentioned that asking proxy consent could be inappropriate, for instance when testing HIV status [90]. Prospective consent [104], e.g., asking informed consent for a study on the treatment of cardiac arrest before the cardiac arrest occurs, is often impossible, because it is not possible to identify these patients prospectively. It has also been argued that deferred consent (i.e., informed consent that is postponed to a later point in time [39, 68, 75, 87, 91, 99]), may not be a legitimate alternative because many patients do not survive their illness [90].

There has been much discussion in the literature about the conditions that should be met when conducting emergency research with a waiver of consent. First, the condition preventing patients from giving informed consent should be a characteristic of the population being studied [63, 68, 75, 91], and the study should be aimed at improving the care of that population [43]. Patients’ previously expressed objections should be respected [75, 87], and both the patient and the data should be appropriately protected [5, 33, 43, 90]. In the absence of consent, it is thought to be particularly important to consider conducting minimal risk research if possible, and to always carefully weigh the risks in relation to the potential benefit to the patient [43, 68, 87, 91]. There should be unanimous agreement among relevant individuals about the importance of the research and the impracticability of informed consent [90]. Further, an independent board should evaluate serious adverse events [99], and IRB approval is necessary [43, 47, 48, 52, 72, 75, 79, 99]. In order to avoid a financial conflict of interest, researchers should not be paid to include patients in the study [99]. An independent physician or advisory board could also consent on the patient’s behalf in such situations [39, 58, 68, 75, 99]. It has further been argued that, for waiver of informed consent to be acceptable, no alternative procedures of equal effectiveness should be available, there should be no intention to give participants feedback of information, and no decisions should be made that affect them [71].

#### Regulations for emergency research with a waiver

Many of the reviewed articles refer to conditions in official regulations [1, 5, 6, 23, 32, 34, 36, 37, 39, 45, 49, 52, 53, 55–57, 60, 61, 64, 65, 68–70, 73, 74, 76, 77, 80–83, 85, 86, 88, 95, 101, 103, 107–112], such as the ‘Final Rule’ of the U.S. Food and Drug Administration (FDA). The FDA amended its informed consent regulations in 1996 in order to ensure that emergency research could be carried out without informed consent in certain situations. The U. S. Department of Health and Human Services (DHHS) adopted these rules, known as the ‘final rule for waiver of informed consent in certain emergency research circumstances’.

According to this ‘final rule’, researchers should consult representatives of the community from which the subjects will be drawn before the start of the study. This provides the opportunity to express one’s views on the proposed study. The IRB must then take these views into account when reviewing the request for the consent waiver. After IRB approval, researchers should publicly disclose the risks and benefits of the study before it starts and after its completion. These risks and benefits should be reasonable in relation to the patient’s condition. It should be made clear that incapacitated individuals may be enrolled without consent from a proxy. The researcher should, however, attempt to contact the patient’s legally authorized representative within the therapeutic window to determine whether they object to participation. If this is not possible, the proxy or patient should be informed as soon as possible, and if the study is still ongoing at that moment, consent should be asked to continue participation. Further, the study’s sponsor should establish an independent data monitoring committee. This committee must exercise oversight of the study, and may recommend continuing, modifying, or stopping the study, dependent on its progress. Moreover, there should be evidence from prior research that the intervention has the potential to benefit patients, while the available treatments are unproven or unsatisfactory. Patients themselves should be in a life-threatening situation that necessitates intervention. It should further be clear that the study couldn’t reasonably be conducted otherwise. For instance, there should be no way in which subjects can be identified prospectively.

In the U.S., government-funded research is further held against an ethical standard known as the ‘common rule’. When it is not practical to obtain consent, the common rule states that an IRB may permit a waiver of consent when the study evaluates public benefit or service programs, procedures for obtaining benefits or services under those programs, possible changes in or alternatives to those programs or procedures, or possible changes in methods or levels of payments for benefits or services under those programs. The Council of Europe has also developed a set of rules on bioethics, which sometimes allows research without consent in those who do not have the capacity to consent [4].

The Declaration of Helsinki also gives exceptions for the rule of informed consent under certain circumstances [4, 39, 45, 54, 99, 113]. A waiver of consent may be granted when subjects are physically or mentally unable to give consent, and when the condition that causes this inability is a necessary characteristic of the research population. Further, attempts to obtain proxy consent should have failed within the therapeutic window. IRB approval should be obtained, and consent to remain in the study should be obtained as soon as possible from the subject or proxy.

#### Other practical problems

There are also other practical problems that authors have suggested may play a role in the argumentation to waive informed consent. A waiver could increase the recruitment rate [5, 10, 38, 114], and decrease the administrative task [16, 45] and resources spent on the study [10]. Further, it may increase the speed at which the study treatment is initiated [37, 38, 68, 76, 87]. Not asking informed consent in these situations may be done under the condition that monitoring of adverse effects takes place, and that this may lead to an intervention [114]. The Zelen design is considered to be an alternative. Another logistical difficulty that has been brought up is time. In case of a public health emergency, such as epidemics of dangerous contagious diseases, there may not be enough time to ask informed consent [25].

Examples of logistical difficulties are cluster randomized trials with large groups of participants [7, 21, 115], or a study on large groups of military personnel going to the Middle-East [62]. This latter example has been explicated by Cummings [62]. The U.S. military wanted to vaccinate 2.4 million troops against inhalation anthrax while it was still an investigational drug, and argued that it was impossible to ask informed consent for this mandatory vaccination. The conditions under which a waiver might be allowed that were mentioned in this paper were the provision of information sheets, careful documentation of whom is given the vaccination and of adverse reactions, and approval of the IRB, FDA commissioner, and the President of the U.S. Further, there should be a public notice of the study.

In cluster randomized trials, the intervention and randomization are on another level than the outcome, for example, in a trial in which a group of physicians receives training and another group does not, and the outcome is a measure of the patients’ health. An example would be a study in which physicians in the intervention arm would, among other measures, be educated about procedures to prevent catheter-related infections, while the outcome, the number of catheter-related infections, is on the level of the patient. It has been argued that informed consent is useless if it is impossible for both patients [21] and healthcare workers to avoid interventions conducted at the level of a complete department or hospital [7].

#### Ethical reasons or objections

Ethical reasons that are proposed to legitimize a consent waiver often relate to, or are consequences of the arguments given in the above paragraphs. Therefore, they can also be divided into the same three categories (decrease of data validity and quality; distress or confusion; and practical problems). It might be unethical to conduct research knowing that the validity of the study results will be compromised. When it is known the results are probably not valid [20, 116], for instance when they are biased [21, 26, 31], it is unethical to spend resources and the participants’ time on research. Further, it might be unethical to distress patients by discussing an experimental treatment with them, after which they are allocated to the control group of the study.

The practical problems with informed consent, especially those in emergency research [1, 34, 39, 49, 63, 84, 85, 99, 108, 117], are thought to lead to ethical problems as well. For instance, it is considered to be unethical to delay treatment initiation because of an informed consent procedure when it is expected that this delay will adversely affect treatment outcome.

However, not all ethical arguments relate directly to arguments in the other categories. In these cases, authors often conduct an ethical analysis, in which they systematically explore the consequences of the different choice options on several fundamental values, such as autonomy, justice, beneficence, or non-maleficence. Commonly, in intervention research, autonomy forms the basis of informed consent. It is argued, however, that respect for autonomy is not valid or is less valid for emergency patients, such as those with traumatic brain injury [34]. Other principles that are relevant for these cases are the prospect of therapeutic benefit, and the protection against potential harm of the intervention. These authors stress the importance of conducting a risk-benefit analysis. A waiver of consent should only be allowed if the risks are acceptable, considering the gravity of the disease [34] and/or the potential therapeutic benefit [43]. Further, it should be ensured that this vulnerable patient group is protected from exploitation due to their incapacitated status [34, 43]. Kompanje further argues that an independent safety committee should be instituted to assess these cases [34].

Risk-benefit analyses sometimes also include benefits on the broader societal level. Some then argue that not asking informed consent may be the most ethically correct thing to do, because it is beneficial for future patients or for society as a whole [3, 35, 39, 41, 54, 57, 63, 76, 84, 85, 108, 118–121]. Informed consent might, for example, prevent or delay progress being made in critical clinical situations which, in turn, could lead to increased mortality or disability, such as in traumatic brain injury [34, 40, 41, 55, 57, 61, 68, 71, 108]. Moreover, if it was not possible to conduct research on emergency care patients without informed consent, patients could be exposed to potentially hazardous effects of invalid and ineffective clinical practice [63]. Evans also noted that it could be argued that patients should participate in scientific research in countries with publicly funded health care, provided the studied treatments are equal in expected treatment outcome [114].

A last group of ethical arguments relates to the idea that it can be ethically unnecessary and/or unreasonable to ask informed consent in specific types of research. Specific conditions, such as the removal of identifiable data [7], the possibility to have data removed on request, or providing information after the end of the study [20] may apply. Main examples are low-risk research types, such as cluster randomized trials [7, 16, 21, 37, 115, 116]. A final argument in this last category is that it is not necessary to obtain consent in the control group and/or the intervention group when the treatments in these arms are routinely used [46, 60].

**Fig. 2:**
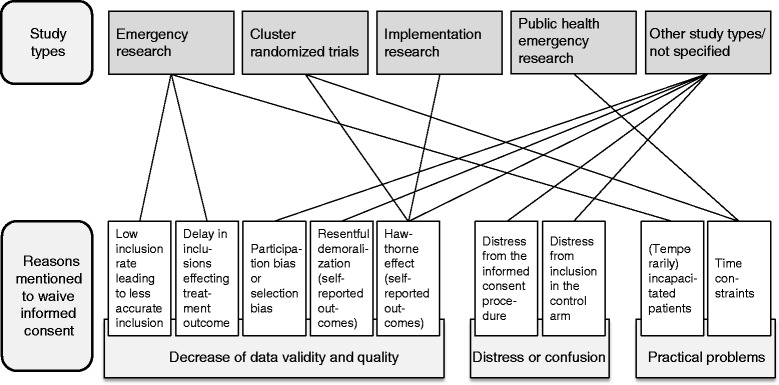
Visualization of the relationship between the different study types and reasons to waive informed consent

Figure 2 visualizes the relationships between different study types and different reasons for not requiring informed consent.

## Discussion

We summarized the different arguments given in the literature to make an exception to the general rule of informed consent. We deliberately did not weigh the number of times arguments were mentioned, because we felt frequency was not related to legitimacy of the argument. However, here we will put the arguments in a broader perspective, in which the frequency of arguments may sometimes be relevant. Three themes emerged from reviewing the literature about reasons to make an exception to the general rule of informed consent for research with an intervention: distress or confusion, practical problems, and data validity and quality. Further, the meta-category of ethical reasons or objections was found to overarch these categories. In the reviewed papers, ethical reasons not to ask informed consent were almost always mentioned alongside arguments in one of the main three categories. For example, in many cases it was argued that it is unethical to conduct research that will yield poor quality data and invalid study results.

Practical arguments against the informed consent requirement come primarily from the field of emergency research, implementation science and proponents of the Zelen design. Key issues in these categories are that research should be of societal importance, should expose participants to no or (relatively) low risks, that IRB approval should be obtained, and that data should be appropriately protected. These issues are more explicitly discussed in the category of practical problems rather than the categories of data quality and participant distress. This might be because emergency research has been discussed more thoroughly than other types of research. We would argue, however, that such conditions, describing when research with a waiver might be conducted responsibly, could also be valuable for researchers in other fields of research in which a waiver might be used.

Data validity and quality is mentioned as the main argument to waive consent for some types of research, such as research with self-reported outcomes or research in which the intervention consists of giving information in such a way that informing people about the study arms would interfere with the outcome. Bias, due to participants’ preference for one of the study arms or due to the higher chance of part of the potential participants (e.g., minority group members) to decline participation, is however the most common argument.

Our review also indicates that not all types of reasons receive equal attention in the literature. Distress or confusion does not seem to be an important category in and of itself. No important research types mentioned only the distress caused by a consent procedure as a reason not to ask informed consent. The distress argument was always given in addition to arguments in one or more of the other categories. Also the argument of practical problems was often given in addition to other arguments, with the exception of very large studies. Importantly, this does not mean that practical problems are not important: for emergency research many authors have raised concerns about practical problems with asking informed consent. Although it is not the only argument, it is the most important argument given by many authors. Arguments such as inferior data quality, the potential distress caused to patients, and serving societal goals, often provide an additional basis for requesting a waiver.

The most often discussed research designs and disciplines can be found in two or three of the categories (Fig. 2). The distress or confusion that a consent procedure might cause to (potential) participants and the practical problems accompanying it most often are used to justify the consent waiver for cluster-randomized trials or (other) studies in implementation science. This is also the case for emergency research and studies for which a Zelen design has been suggested, with the addition of a decrease in data quality. Professionals thus saw different types of reasons why it is undesirable to always ask informed consent in these research situations. It seems clear that the likelihood of the (perceived) need for an informed consent waiver in a specific type of research increases when the difficulties related to informed consent span different categories. Although it should be no surprise that more reasons take up more space in the literature, it seems equally likely that when there are several types of difficulties with asking informed consent, more researchers find a waiver acceptable, for instance because it becomes more difficult to find other solutions.

Ultimately, it is the researcher’s responsibility to provide sufficient argumentation for waiving informed consent, and that of the IRB to weigh the arguments carefully before granting such a waiver for any given project. This often involves subjective judgment since, to the best of our knowledge, no criteria have been put forward that indicate the extent to which any argument (e.g., patient distress, study bias, etc.) is sufficiently strong to warrant a consent waiver.

### Usage of consent waivers in practice

Statements found in the literature that researchers only consider a consent waiver after careful consideration of different factors corresponds with our own experience. As noted in the introduction, we conducted a study that inspired us to review reasons to waive informed consent. In this study, we compared three different consent procedures for the use of residual tissue for scientific research in a randomized controlled trial [8]. During this study, it appeared to be very confusing for patients to be asked informed consent for an intervention study with three arms comparing different consent procedures. Therefore, when we were planning a larger study to replicate our initial findings, we requested and were granted a consent waiver. This was based on several arguments, including the fact that patients could be confused by being asked for informed consent within the context of an informed consent intervention, that it was important for the study procedures to mirror actual medical practice, and that the intervention was non-invasive.

Not all of the reviewed research types and designs find equal support in the medical research community. The Zelen design is controversial, most notably because it is considered unethical to observe people who are unaware of being randomized to a study, and because it requires a larger sample size than regular randomization due to patients not willing to receive the treatment of the intervention arm (e.g. [42, 45, 122]). This research design is therefore seldom used in practice [28].

However, arguments in favor of waiving consent in some specific situations in emergency research were sufficiently convincing to be incorporated in official rules and regulations. For the Declaration of Helsinki, the addition of this waiver in the fifth edition in 2000 represented an important change in the informed consent requirements [54].

### Future research

Future research may benefit from a focus on two topics. First, the current literature on consent waivers mainly focuses on Western countries. More research is needed on exceptions to the general rule of informed consent in non-Western cultures. Secondly, not much is known of the patients’ views on waiving consent. Although some research has been conducted, these studies all concern using a waiver for emergency research (e.g. [123, 124]). In 2008, Lecouturier et al. [125] concluded in their review on participants’ views about research without informed consent specifically in the emergency setting that insufficient information was available to draw any firm conclusions.

## Conclusions

What does this all mean for researchers preparing an intervention study with a consent waiver? First, it is important to keep in mind that situations justifying consent waiver are always an exception to the rule. Informed consent should always remain the standard in research with an intervention. Moreover, researchers always need to comply with the rules and guidelines applicable in their country, institute and research field. When these criteria are fulfilled, Fig. 2 may be used to oversee the types of reasons legitimized by others in different types of studies. These reasons can be used by research groups to form their own opinions on whether, and under which conditions they find waiving informed consent in their own study appropriate. Importantly, researchers should think through whether the arguments they believe to be legitimate are valid for all parts of an informed consent procedure. For instance, in some cases, waiving consent for an intervention could be appropriate, whereas consent, deferred consent, or deferred proxy consent, may be asked for the use of a patient’s data. This distinction is not commonly made in the literature we reviewed.

We wish to stress that the reasons to waive consent mentioned above are not the only reasons that could justify a consent waiver. Other situations not discussed in the literature may arise, and the reasons to waive consent mentioned in this paper may serve as examples that are acceptable according to at least part of the research community. Further, the above review is not an ethical analysis. Moreover, different cultural settings or personal believes may differ. Therefore, some readers may disagree with waiving informed consent in some of the studies mentioned in this paper. Importantly, researchers should also take the conditions under which such an exception is justified into consideration, such as additional privacy protection measures. Although ultimately it is the IRB that will approve or disapprove requests to waive standard informed consent procedures, it behooves researchers to discuss their argumentation with relevant stakeholders, including other scientists, ethicists, physicians, and patient representatives before submitting a formal request to an IRB. Ultimately, it is the creation of consensus among relevant stakeholders that will legitimate any decision to deviate from the standard rules and regulations governing the conduct of medical research.
